# Investigation to determine staff exposure and describe animal bite surveillance after detection of a rabid zebra in a safari lodge in Kenya, 2011

**DOI:** 10.11604/pamj.2014.19.10.3434

**Published:** 2014-09-07

**Authors:** Mark Obonyo, Wences Arvelo, Samuel Kadivane, Moses Orundu, Emily Lankau, Francis Gakuya, Peninah Munyua, Jane Githinji, Nina Marano, Kariuki Njenga, Jared Omolo, Joel Montgomery

**Affiliations:** 1 Kenya Field Epidemiology and Laboratory Training Program, Ministry of Health, Nairobi, Kenya; 2 Global Disease Detection, US Centers for Disease Control and Prevention, Kenya; 3 Loitoktoik District Hospital, Ministry of Public Health and Sanitation, Kenya; 4 Division of Global Migration and Quarantine, US Centers for Disease Control and Prevention, Atlanta, USA; 5 Epidemic Intelligence Service, US Centers for Disease Control and Prevention, USA; 6 Kenya Wildlife Service, Ministry of Forestry and Wildlife, Kenya; 7 Central Veterinary Laboratory, Ministry of Livestock Development, Department of Veterinary Services, Kenya

**Keywords:** Rabies, outbreak, epidemiology, East Africa

## Abstract

**Introduction:**

Rabies is a fatal viral infection, resulting in >55,000 deaths globally each year. In August 2011, a young orphaned zebra at a Kenyan safari lodge acquired rabies and potentially exposed >150 tourists and local staff. An investigation was initiated to determine exposures among the local staff, and to describe animal bite surveillance in the affected district.

**Methods:**

We interviewed lodge staff on circumstances surrounding the zebra's illness and assessed their exposure status. We reviewed animal bite report forms from the outpatient department at the district hospital.

**Results:**

The zebra was reported bitten by a dog on 31^st^ July 2011, became ill on 23^rd^August, and died three days later. There were 22 employees working at the lodge during that time. Six (27%) had high exposure due to contact with saliva (bottle feeding, veterinary care) and received four doses of rabies vaccine and one of immune-globulin, and 16 (73%) had low exposure due to casual contact and received only four doses of rabies vaccine. From January 2010 to September 2011, 118 cases of animal bites were reported in the district; 67 (57%) occurred among males, 65 (57%) in children <15 years old, and 61 (52%) were inflicted in a lower extremity. Domestic and stray dogs accounted for 98% of reported bites.

**Conclusion:**

Dog bites remains the main source of rabies exposure in the district, but exposure can result from wildlife. This highlights the importance of a one health approach with strong communication between wildlife, veterinary, and human health sectors to improve rabies prevention and control.

## Introduction

Rabies virus (family *Rhabdoviridae*, genus *Lyssavirus*) causes an acute and fatal encephalomyelitis characterized by apprehension, headache, fever, malaise, and sensory changes, such as paresthesia at the site of an animal bite or even the entire extremity[1]. Worldwide >10 million persons are potentially exposed to the rabies virus each year, resulting in an estimated 55,000 deaths. Over 95% of mortality due to rabies infection occurred in Africa and Asia [2, 3]. Animal reservoirs include carnivores and bats [4–7].

Rabies transmission is usually through saliva via the bite of an infected animal, though infection can also occur through scratches. The incubation period in humans is highly variable, ranging between three to eight weeks, but in some cases could be as short as a few days or as long as several years depending on site of the bite, severity of wound, and amount and type of rabies virus involved[1]. The viral shedding period is known in dogs, cats, and ferrets, and is usually three to seven days before onset of clinical manifestations of rabies and throughout the course of the disease. Longer shedding periods of 10-14 days prior to the onset of symptoms have been observed with certain canine rabies virus variants in experimental infections, but these are the exception [8]. Viral excretion in other animals is highly variable, for example, in one study in bats viral shedding for 12 days before evidence of illness was documented[9]. Thus, infectious periods for wildlife are typically conservatively estimated as at least 10-14 days before onset of illness.

Management after potential exposure to the rabies virus consists of post exposure prophylaxis (PEP)[10]. The level of exposure risk guides PEP recommendations, as per the World Health Organization (WHO) guidelines [3, 11]. In general, rabies PEP consists of thorough wound cleansing and infiltration of the site bite with human or equine rabies immune globulin (RIG) immediately after the exposure. This is followed by five intramuscular doses of rabies vaccination on days 0, 3,7,14 and 28 after [12]. RIG is administered to immediately neutralize the virus at the bite site and bridge the immunological gap after administration of the rabies vaccine.

In Kenya, rabies is a significant public health concern that in some districts results in approximately three deaths per 100,000 persons annually [13–15]. The majority of human rabies cases are acquired from dog bites. The annual incidence of animal bites in humans in Kenya is estimated as 234 per 100 000 persons [16]. In Kenya, animal bite surveillance is conducted passively[13]. The main incentive for reporting animal bites is the provision of free rabies vaccine by the government healthcare system. Exposures to rabies from wildlife bites sometimes occur, but such events are rarely reported and the role of wildlife in the epidemiology of the disease is not well documented[15].

On July 31, 2011, a zebra foal housed at a Kenyan safari lodge in Loitoktok District was bitten by a feral dog. Approximately one month later, the zebra died after experiencing a neurological illness consistent with rabies. Brain tissue was submitted to the Central Veterinary Laboratories (CVL) in Kabete on August 30, 2011. The rabies diagnosis was laboratory confirmed by a fluorescent-antibody test. There was an immediate health concern given that both staff and guests at the lodge had opportunities for physical contact with the zebra. The lodge distributed a statement by email to guests from several countries around the world, alerting them of the possible exposure to the rabies virus, and recommending individuals to seek clinical consultation for further guidance. On September 1, 2011, the US Centers for Disease Control and Prevention (CDC) received reports from two State Health Departments regarding need for PEP among American tourists that had visited the lodge [17]. The CDC immediately contacted the Kenyan Ministry of Public Health and Sanitation, and the event was reported to the WHO. A multinational investigation among US and other international tourists who visited the affected lodge was initiated to ensure that travelers potentially exposed to the rabies virus received appropriate rabies PEP. On September 12, 2011, we conducted an investigation to better characterize the events surrounding the zebra's illness and death, to determine the levels of potential exposure to rabies virus among local staff and other animals housed in the lodge stables, conduct a site inspection, and to evaluate animal bite surveillance in the district.

## Methods

### Site Investigation

We interviewed lodge staff concerning the zebra's clinical history, their exposures to the zebra, and rabies vaccination status of the staff. Since the zebra was housed within the stables compound, we conducted a site inspection of the stables to better assess and determine the potential level of exposure to rabies virus among local staff working at the lodge and in the stables, as well as other animals housed in the stables compound.

### Animal bite surveillance

We conducted a retrospective review of animal bite cases reported by the ambulatory department of the Loitokitok District Hospital between January 2010 and September 2011. Level of district preparedness was assessed based on the WHO report on Expert Consultation on Rabies. WHO. A case of animal bite was defined as any bite injury from any animal to a resident of Loitokitok District. Data on animal bites were recorded on a standard case report form for animal bite surveillance developed by the Kenyan Ministry of Public Health and Sanitation, and included demographic information, date of bite, type of animal that bit the individual, location of bite, and rabies vaccine status after the bite. The reporting forms were completed by the physician or nurse treating the patient. We recorded the data using Microsoft Excel 2007 (Microsoft, Seattle, WA, USA), and analyzed using Epi Info version 3.5.3 (CDC, Atlanta, GA, USA) and Microsoft Excel 2007. We calculated proportions for categorical variables and means and medians for continuous variables.

### Ethics

This investigation was considered a public health response to an acute event by the Kenyan Ministry of Public Health and Sanitation, and as such, did not require review by an institutional review board. The investigational protocol was approved by the Kenyan Ministry of Public Health and Sanitation. During the site inspection informed consent was not obtained from the staff, as these interviews were conducted strictly as a public health response activity. Measures were taken to assure confidentiality of the information provided during these interviews. For the review of animal bite surveillance, data analyzed for this purpose were collected as part of routine surveillance conducted by the Ministry of Public Health and Sanitation, and did not include any personal identifying information. Measures were taken to assure collected data were properly stored and secured.

## Results

### Lodge investigation

A one month old zebra was adopted by the mobile safari company associated with the safari lodge in January 2011. The zebra was kept within the horse stables and it was free to mingle with the horses and other stable animals, staff, and lodge guests. The zebra had not been vaccinated against rabies. The zebra had become an attraction to tourists visiting the mobile safari and the lodge, and many participated in feeding, petting, and taking photographs with the zebra. Stable keepers reported that the zebra had been bitten on the muzzle by a feral dog on July 31, 2011. On August 23, 2011 the zebra began to show symptoms that included fever and inability to feed that eventually progressed to erratic behavior described by the staff as aggressiveness, trying to bite persons and horses, becoming unusually attached to human handlers, and hyperactivity. The zebra died on August 26, 2011. The 36 staff members who had contact with the zebra were all taken to a nearby private hospital on September 1, 2011, where exposure status to rabies virus was assessed. Exposure risk was classified either as low or high risk depending on the nature of interaction with the zebra during the estimated infectious period. Although the duration of viral shedding for zebras is unknown, the infection period was estimated to be 14 days before illness until death or from August 10^th^ to August 26^th^ [17]. High risk exposures to the zebra during the infectious period among humans included petting, feeding, grooming, and conducting necropsy on the dead zebra. Six (18%) employees were considered having a high risk exposure status such as hand feeding the zebra, whereas 28 (82%) employees had low risk exposure status, which included visiting or working in the stables or lodge. Employees with low exposure risk were initiated on the five vaccine dose regimen, with the first vaccine given at the time of presentation followed by additional doses on days three, seven, 14 and 28 after presentation. Those with high risk received the same regimen plus one dose of RIG on the first day of presentation. There were no observed human cases as a result of these exposures.

### Site inspection

The stables housed 36 horses, one dog and one cat. The stable compound was clean and well kept. Each horse was kept in a separate stall where it was fed separately from other animals. Each stall had a wooden door which was locked when a horse was inside. The stable yard was fenced with a barbed wire, but there was the possibility that small animals like wild or feral dogs, could gain access. However, apart from the feral dog that bit the zebra, there were no additional reports of feral animals entering the stable compound. All the animals housed in the stable had up-to-date vaccination against rabies, and none of the animals developed any symptoms suggestive of rabies.

### Animal bite surveillance

From January 2010 to September 2011, 118 cases of animal bites were reported to the ambulatory department of Loitokitok District Hospital. An average of seven bites was reported per month (range: 0-12 bites/month). The number of bites was evenly distributed throughout the year with slight peaks in February 2010 and June 2011. There were no records of animal bites reported between January and April 2011, since during this period there were no rabies vaccines available at the district hospital and all patients presenting with animal bites were referred elsewhere. Among the 118 reported cases, 67 (57%) occurred in males. Ages were recorded from 114 of the cases. Median age was 13 years (range: 1 to 84 years). Fifty-four (47%) bites occurred among those aged 5-14 years; 11 (10%) were children <5 years old (Table 1). Geographic clustering was observed among cases, with bites occurring more frequently among residents of villages less than 10 Km from the district hospital, in areas classified as urban and peri-urban. These locations accounted for 99 (87%) of all the reported cases in the district. Sixty-one (52%) bites occurred on the lower extremity (Table 2). In the age group of 5-14 years, six (83%) were reported on the shoulder, back, and chest. The five (100%) cases of bites to the head, face, and neck occurred in children aged less than five years old. The majority (98%) of the bites were from dogs, of which 96 (83%) were owned dogs, while 20 (17%) were considered feral. Cats accounted for the remaining two (2%) bites. All of the cases were reported to have received rabies vaccine. Sixteen (14%) of the animal bite victims received five vaccination doses, 15 (13%) received four doses, 63 (53%) received three doses, 15 (13%) received two doses, and nine (8%) received one dose. None were reported to have received RIG and none if the vaccinated patients were reported to have developed rabies. On the level of district preparedness, we identified an insufficient supply of rabies vaccine for both human and animals, with stock-outs occurring over long periods in the district hospital. There was also no RIG, as the use of RIG is not included as a treatment recommendation within the current national guidelines. There was also limited communication and information sharing between the officials from the Ministry of Livestock, Kenya Wildlife Services, and Ministry of Public Health and Sanitation in regards to rabies control and animal bite surveillance at the district level. There was no systematic strategy for dog population management. Vaccination of dogs was done sporadically by the District Veterinary Officer.

**Table 1:** Number of reported animal bites by age group and sex, Loitokitok District 2010/2011 (N=114*)

Characteristics	Reported Bites n (%)
**Sex**	
Male	65 (57)
Female	49 (43)
**Age group < 5 years**	
Male	7 (11)
Female	4 (8)
**Age group 5-14 years**	
Male	36 (55)
Female	18(37)
**Age group 15-24 years**	
Male	6 (9)
Female	6 (12)
**Age group < 25 years**	
Male	16 (25)
Female	21 (43)

* Four patients did not have their ages recorded

**Table 2:** Distribution of dog bites by anatomical site and age group, Loitokitok District, 2010/2011

Body site	Age group (years)	Reported Bites n (%)
Hands, finger		
	<5	4 (21)
	5 - 14	7 (37)
	15 - 24	2 (11)
	>25	6 (32)
Head, face, neck		
	<5	2 (40)
	5 - 14	3 (60)
	15 - 24	0
	>25	0
Lower leg, foot, toe		
	<5	4 (7)
	5 - 14	24 (41)
	15 - 24	6 (10)
	>25	25 (42)
Shoulder, back, chest		
	<5	1 (14)
	5 - 14	6 (86)
	15 - 24	0
	>25	0
Hips, buttocks, thigh, knee		
	<5	0
	5 - 14	13 (59)
	15 - 24	4 (18)
	>25	5 (23)
Not indicated		
	<5	0
	5 - 14	1 (50)
	15 - 24	0
	>25	1 (50)

## Discussion

This investigation highlighted the fact that in Kenya human exposure to the rabies virus can occur from contact with wildlife. However, such exposures are rarely reported and not well documented in Kenya. Wildlife in Kenya accounts for less than one percent of reported rabies cases in animals, suggesting that rabies in wildlife is underreported. Despite a large presence of wildlife in the country, the role of wildlife in the epidemiology of rabies is poorly defined since only a limited number of specimens from wild carnivores, bats and other wildlife species are received at CVL for rabies virus testing each year[4]. This investigation documented unique circumstances of human exposure to a wild animal demonstrating that wildlife can result in a potential source for rabies virus exposure among persons residing or visiting Kenya.

Our evaluation of animal bite surveillance in the affected district showed that bites from owned dogs continue to be a major public health problem in Kenya, leading to potential rabies virus exposures in the population. These findings were similar to previous studies showing that dog bites have become a major public health problem worldwide, particularly among male children <15 years who have a higher risk of being bitten by dogs [18–22]. Although most animal bites occurred on the extremities, all bites reported on the head were among children <5 years old. Young children may be predisposed to bites to the head, face, or neck due to their height and proximity to the animals. Bites to the head require especially prompt medical intervention because victims of these bites are vulnerable to more rapid clinical progression to rabies due to high density innervations of these tissues and proximity to the central nervous system [23].

Additionally, we also found a lack of consistent vaccine availability in the district hospital. This disruption in rabies vaccine supply not only resulted in inadequate management of persons potentially exposed to the rabies virus, but also led to disruption in the reporting of animal bites by healthcare workers, as reporting is entirely linked to vaccine provision in government health facilities. Moreover, there is no availability of RIG in public health facilities in Kenya, resulting in an inability to provide complete and adequate rabies PEP to victims of animal bites according to WHO guidelines. A number of PEP failures have been associated to insufficient or lack provision of RIG, as the immunoglobulin is critical in immediately neutralizing the virus prior to developing an immunological response after receiving the vaccine. This emphasizes the importance of RIG for the most effective rabies prevention following exposure to the virus [10, 12]. Surveillance forms for animal bites did not include additional information such as nature and severity of the wound, other treatments given including RIG, details of biting animal such as age, breed or species, circumstances resulting in the bite, rabies vaccination history of the animal, outcome of the animal, and whether the animal was tested for rabies virus.

Effective prevention and control of rabies requires close collaboration with partners in the human and animal health sectors. Animal bite surveillance on the human side should inform proper dog population management and vaccination against rabies. Vaccinating owned dogs against rabies has been shown to be the most cost-effective intervention in the medium and long term; costs are typically recouped within 5-10 years, mainly through decreased expenditure on human post-exposure treatment [10, 16, 24].

## Conclusion

Though rabies virus exposures from owned dog bites continue to be a challenging problem in Kenya, the large-scale exposure of staff and guests at a safari lodge highlighted that non-canine wildlife species can also become infected with rabies virus and pose an exposure risk. To ensure effective control of rabies in Kenya, a one health approach with strong linkages and communications between the public health and veterinary officials, as well as officials from the wildlife services, is critical for prompt and adequate rabies diagnosis, surveillance, prevention and control. There is a need for increased reporting of animal bites and sharing of data regarding suspected rabies cases between public health, wildlife and veterinary authorities. There should also be deliberate efforts to ensure inclusion of RIG within national guidelines for the management of potential rabies virus exposures, as well as an adequate and uninterrupted supply of rabies PEP in all government health facilities. Rabies is a preventable disease, but effective prevention requires a multisectoral approach for adequately addressing the various components within the human and animal sectors in an effort to control and prevent rabies in Kenya.
